# The Effect of Geometrical Overlap between Giant Magnetoresistance Sensor and Magnetic Flux Concentrators: A Novel Comb-Shaped Sensor for Improved Sensitivity

**DOI:** 10.3390/s22239385

**Published:** 2022-12-01

**Authors:** Prabhanjan D. Kulkarni, Hitoshi Iwasaki, Tomoya Nakatani

**Affiliations:** Research Center for Magnetic and Spintronic Materials, National Institute for Materials Science, 1-2-1, Sengen, Tsukuba 305-0047, Japan

**Keywords:** magnetoresistive sensors, giant magnetoresistance, magnetic flux concentrator, magnetic circuit, finite element method simulation

## Abstract

The combination of magnetoresistive (MR) element and magnetic flux concentrators (MFCs) offers highly sensitive magnetic field sensors. To maximize the effect of MFC, the geometrical design between the MR element and MFCs is critical. In this paper, we present simulation and experimental studies on the effect of the geometrical relationship between current-in-plane giant magnetoresistive (GMR) element and MFCs made of a NiFeCuMo film. Finite element method (FEM) simulations showed that although an overlap between the MFCs and GMR element enhances their magneto-static coupling, it can lead to a loss of magnetoresistance ratio due to a magnetic shielding effect by the MFCs. Therefore, we propose a comb-shaped GMR element with alternate notches and fins. The FEM simulations showed that the fins of the comb-shaped GMR element provide a strong magneto-static coupling with the MFCs, whereas the electric current is confined within the main body of the comb-shaped GMR element, resulting in improved sensitivity. We experimentally demonstrated a higher sensitivity of the comb-shaped GMR sensor (36.5 %/mT) than that of a conventional rectangular GMR sensor (28 %/mT).

## 1. Introduction

Giant magnetoresistance (GMR) devices with current-in-plane geometry provide magnetic sensors for various applications such as gradiometers and magnetic encoders owing to their large output voltage, controllable field range and reliability [1,2,3,4]. The GMR sensors are promising not only for general purpose magnetic sensors but also for high-sensitive magnetic sensors to detect small magnetic fields, e.g., biomagnetic fields or stray fields from magnetic nanoparticles for biomedical applications, in combination with magnetic flux concentrators (MFCs) [5,6]. MFCs are made of soft-magnetic film/foil/bulk materials that concentrate magnetic flux into magnetic sensor elements, e.g., Hall [7,8], anisotropic magnetoresistance [9], GMR [3,10,11], and tunnel magnetoresistance [12,13] sensors. Typically, larger MFCs in size provide more effective magnetic flux concentration; however, the large footprint of MFC is a tradeoff for sensor applications. In magnetic sensors with MR element, generally the size of the MFC is larger than the sensing element and acts as a limiting factor to the spatial resolution of a sensor. Therefore, it is important to develop the sensors with smaller MFCs. Various methods have been implemented to improve the sensitivity of magnetic sensor with an on-chip MFC and MR element. In 2004, Pannetier et al. proposed a GMR sensor with a superconducting MFC loop for a femto-tesla field measurement at low temperature [14]. Other reports show that the detectivity of MR sensor can be improved by modulating the magnetic field to higher frequency using MFCs or MR elements with microelectromechanical systems-based (MEMS) mechanisms [15,16,17]. Recently, Kikitsu et al. [18] have implemented highly sensitive GMR sensors (detectivity~13 pT/Hz^1/2^ at 100 Hz) with on-chip MFCs and an external ac modulation field in a magnetic field microscope and observed a better spatial resolution in comparison with magneto-impedance sensors.

**Figure 1:**
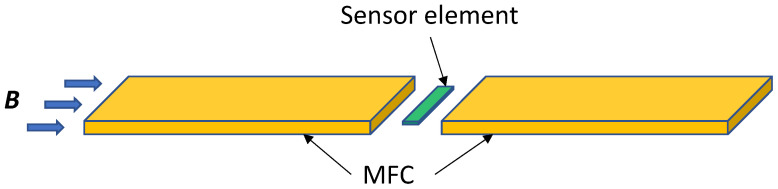
Schematic representation of a typical magnetic sensor with MFCs.

Figure 1 shows a schematic configuration of a GMR sensor with MFCs. The GMR element is typically a rectangular-shaped spin-valve (SV) film with a width of a few micrometers to a few tens micrometers patterned by the microfabrication technique. A pair of MFCs, typically made of permalloy, are placed next to the GMR element. In principle, a larger relative permeability (*μ*_r_) of the MFC material and a narrower gap between the MFCs create a lower magnetic reluctance path for magnetic flux. Hence, MFCs concentrate external magnetic flux into the sensor element placed between the gap of the MFC. The efficiency of an MFC is often described by MFC gain, which is defined as $B_{\mathrm{sen}}^{\;\mathrm{MFC}}{/B}_{\mathrm{sen}}^{\;\mathrm{noMFC}}$, where $B_{\mathrm{sen}}^{\;\mathrm{MFC}}$ and $B_{\mathrm{sen}}^{\;\mathrm{noMFC}}$ are the magnetic flux densities inside the sensor element with and without MFC, respectively. However, for magnetoresistive (MR) sensors consisting of MFCs, the net magnetic reluctance of the whole sensor system depends not only on the *μ*_r_ of the MFCs but also on that of the sensor element. Therefore, it is important to design the magnetic circuit of sensors with MFCs considering the magnetic properties and geometrical parameters (e.g., shape, gap, width, and length) for both MFC and magnetic free layer (FL) of the MR element. Although there are studies on the effects of the magnetic properties and geometrical parameters of MFCs on the MFC gain, [13,19,20,21,22,23,24] discussions including the magnetostatic coupling between MFCs and FL are limited.

In this work, we studied the effect of the geometrical configuration of the GMR element and MFCs on the sensitivity of the GMR sensor. We simulated the distribution of magnetic flux in the GMR elements with two kinds of shape: a conventional rectangular and a comb shape. We also studied the sensitivity of these sensors experimentally. We found that the geometrical overlap between the rectangular GMR element and MFCs can give either enhancement or reduction of the GMR ratio depending on the degree of overlap. For the comb-shaped GMR elements, however, we obtained an improved sensitivity compared to the case with the rectangular GMR elements.

## 2. Magnetic Circuit in the MFC–FL System

**Figure 2:**
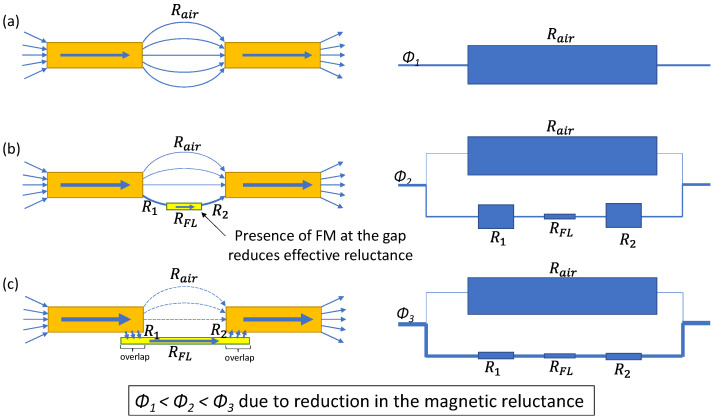
Schematic diagrams showing the effect of the present of an FL on the magnetic reluctance to the magnetic flux flowing through the sensor: (**a**) without FL, (**b**) with FL, and (**c**) with overlap between the MFC and FL.

Since MR sensor elements consist of ferromagnetic FLs with relatively large *μ*_r_, the magneto-static coupling between the FL and MFCs influences the magnetic flux density (*B*) in the FL. This is qualitatively explained in Figure 2. As shown in Figure 2a, when only a pair of MFCs presents without a MR element in the gap, the magnetic flux (*Φ*_1_) through the MFCs is determined by the magnetic reluctances of the MFCs and the air gap (*R*_air_). When a FL with a magnetic reluctance of *R*_FL_ is placed in the gap (Figure 2b), the magnetic reluctance between the MFCs is reduced compared to *R*_air_ because of the lower magnetic reluctance of *R*_1_ + *R*_FL_ + *R*_2_ than *R*_air_, where *R*_1_ and *R*_2_ are the magnetic reluctances of the spacing between the FL and MFC. The FL provides a low magnetic reluctance path due to its large *μ*_r_ (≫1). Therefore, the presence of FL in the MFC gap not only increases the magnetic flux flowing through itself, but also increases the total magnetic flux flowing through the whole sensor (i.e., *Φ*_2_ > *Φ*_1_ in Figure 2). Therefore, by increasing the magneto-static coupling between MFC and FL, the effect of the MFC, i.e., the sensitivity of the sensor, improves. As shown in Figure 2c, by introducing an overlap between the FL and MFCs with a small vertical spacing for electrical separation between them, the magneto-static coupling between the FL and MFCs is further enhanced.

## 3. Methods

### 3.1. Finite Element Method Simulations

**Figure 3:**
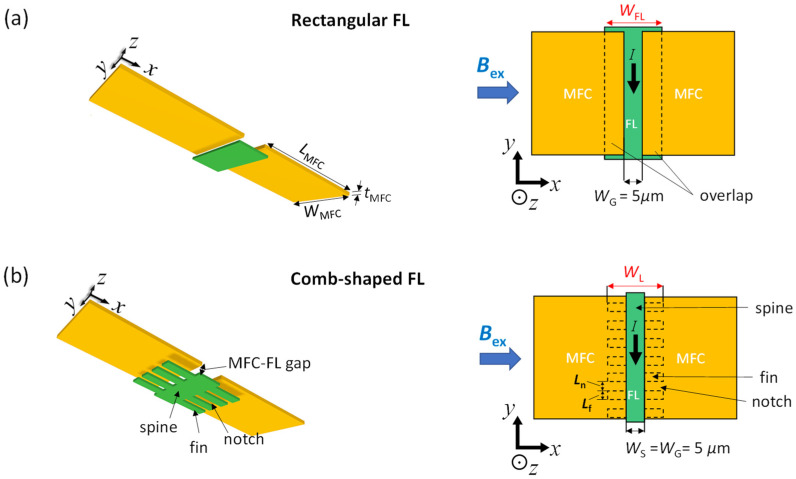
3D and 2D schematics of the GMR sensors with (**a**) a conventional rectangular FL and (**b**) a comb-shaped FL. The schematics are not proportional to actual lengths. MFC-FM overlaps exist for *W*_FL_, *W*_L_ > *W*_G_.

We simulated the distributions of *B* and electric current (*I*) in sensor systems composed of a GMR element and a pair of MFCs using the finite element method (FEM) with the AC/DC module of COMSOL Multiphysics^®^ software [25]. The region surrounding the GMR element and MFCs was defined as “air” (i.e., magnetic permeability of *μ_0_*) with boundary condition at infinity. The magnetic field generated from an electric current *I* flowing through the GMR element was not considered in the analysis. For the GMR element, only the FL was considered because the magnetizations of the other ferromagnetic layers (i.e., pinned layer (PL) and reference layer (RL)) are fixed. Figure 3 shows the three-dimensional (3D) and two-dimensional (2D) (in the xy-plane) schematics of the simulated structures consisting of the MFCs and FL. We simulated two types of the shape of the FL; a conventional rectangular (Figure 3a) and a comb shape (Figure 3b). Both the rectangular and comb-shaped FLs are located in the gap between the MFCs with a spacing of 0.1 μm in the z-direction, which is for the electrical separation between them. In the comb-shaped FL, the “fins” are separated by the “notches” and connected to the central “spine” of the FL. The lengths of the fins and notches in the y-direction were defined as *L*_f_ and *L*_n_, respectively. *I* was flown in the y-direction.

For both the rectangular and comb-shaped FLs, the shape and size of the MFC were fixed to a cuboid with a length of *L*_MFC_ = 1000 μm, a width of *W*_MFC_ = 100 μm, and a thickness of *t*_MFC_ = 1 μm. Typically, a large (MFC length/MFC gap) ratio (greater than 100) is desirable for a higher MFC gain. At the same time, if the MFC gap is comparable to or less than *t*_MFC_, it may increase the flux leakage between the two MFCs. Considering these aspects, the gap between MFCs was set to *W*_G_ = 5 μm in the present study. Note that MFCs do not necessarily have to be cuboids but can assume various shapes, such as trapezoids, and taper in thickness [23]. However, in this work we simulated only for cuboidal MFCs for simplicity. The thickness of the FL was fixed to 10 nm for both the rectangular and comb-shaped FLs. Although the actual thickness of the FL in the experiments was 5.5 nm, the choice of 10 nm for the FL thickness was limited by the finite memory size of the computer used for the FEM simulations. For the rectangular FLs, the MFC and FL overlap (hereafter, MFC–FL overlap) when the width of the FL (*W*_FL_) was larger than *W*_G_. For the comb-shaped FLs, the width of the spine (*W*_S_) was kept the same as *W*_G_ (=5 μm). Therefore, the fins of the comb-shaped FL provided an MFC–FL overlap. The total width of the comb-shaped FL including the spine and fins was defined as *W*_L_. The relative permeabilities of the FL ($\mu_{r}^{FL}$) and MFC ($\mu_{r}^{MFC}$) were assumed to be 500 and 2000, respectively, based on the experiments described below. The effects of *W*_FL_ (for the rectangular FL), *W*_L_, *L*_f_, and *L*_n_ (for the comb-shaped FL) on *B* inside the FLs were simulated under the application of a uniform external *B* (*B*_ex_) in the x-direction.

### 3.2. Experimental Procedures

We deposited the SV structure on a 2 cm × 2 cm substrate with a confocal sputtering system with a rotating substrate. Then, the SV devices and corresponding MFCs were fabricated on the same piece of substrate. This allowed us to keep the fabrication conditions the same for all devices, which included (i) the temperature, field, and time of magnetic annealing; (ii) the microfabrication process (photolithography, ion-milling etc.); and (iii) the misalignment between MFC and GMR patterns.

**Figure 4:**
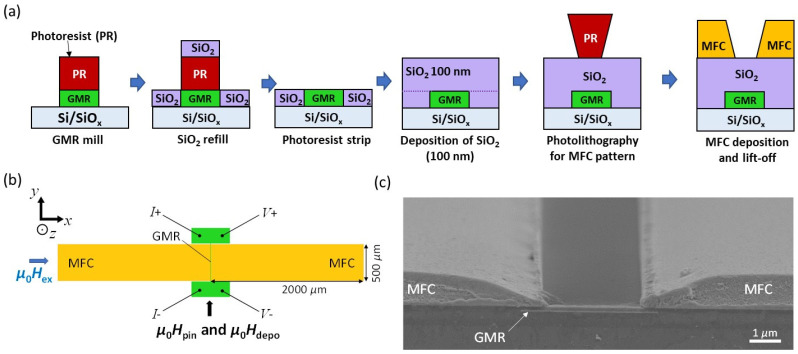
(**a**) Fabrication process flow of the GMR sensors with MFCs. (**b**) Schematic plan view and (**c**) cross-sectional SEM image of the GMR sensors.

First, a bottom-pinned SV with the structure of Ta (2)/Ru (2)/IrMn (6)/Co_50_Fe_50_ (2.5)/Ru (0.8)/Co (3)/Cu (3)/Co (0.5)/Ni_80_Fe_20_ (5)/Ru (4) (thickness in nm) was deposited on a thermally oxidized Si substrate by a DC magnetron sputtering at room temperature. The Co_50_Fe_50_ (2.5), Co (3), and Co (0.5)/Ni_80_Fe_20_ (5) layers acted as PL, RL, and FL, respectively. The film was annealed at 250 °C for 1 h under a magnetic field of 0.7 T. The unpatterned FL showed a saturation magnetic flux density (*B*_s_) of 1.1 T, a magnetic coercivity (*μ*_0_*H*_c_) of 0.2 mT, and a saturation field (*μ*_0_*H*_s_) of 2.15 mT along the hard axis perpendicular to the pinning direction; thus, *μ*_r_ = 512. The microfabrication process of the GMR sensors with MFCs is shown in Figure 4a. First, the GMR elements with rectangular and comb shapes were patterned by a combination of photolithography and Ar ion milling. The GMR elements were patterned in such a way that their widths (i.e., *W*_FL_ and *W*_L_) were aligned perpendicular to the pinning direction (*μ*_0_*H*_pin_) as indicated in Figure 4b. Before photoresist was stripped for the milling of the GMR elements, a SiO_2_ insulation layer of the same thickness as the total GMR SV stack (~30 nm) was deposited, by which the surface of the sample remained flat after the photoresist was stripped. Then, another SiO_2_ layer with a thickness of 100 nm was deposited on the whole surface of the substrate to electrically insulate the GMR elements from the MFCs.

Next, the MFCs with a rectangular shape were fabricated by a lift-off process using a 3 μm-thick negative photoresist (ZEON Corporation, ZPN 1150) with an undercut (readers may refer to ref. [26] for general information on the use of a negative photoresist in the lift-off process). The size of the MFCs was 2000 μm × 500 μm as shown in Figure 4b. The MFCs were sputter-deposited as a multilayer of Conetic alloy (Ni_77_Fe_14_Cu_5_Mo_4_ (at. %)) and Ta: [Ta (5 nm)/Ni_77_Fe_14_Cu_5_Mo_4_ (50 nm)]_×20_/Ta (5 nm) under a magnetic field (*μ*_0_*H*_depo_) of 10–20 mT to induce uniaxial magnetic anisotropy. *μ*_0_*H*_depo_ was kept parallel to *μ*_0_*H*_pin_ (i.e., *y*-axis). The multilayering of the Ni_77_Fe_14_Cu_5_Mo_4_ films with the Ta intermediate layers resulted in a soft-magnetic MFC film with *B*_s_ = 0.6 T, an anisotropy field (*μ*_0_*H*_k_) of 0.28 mT, a hard axis *μ*_0_*H*_c_ of 0.01 mT; thus, *μ*_r_ = 2140. Figure 4c shows the cross-sectional scanning electron microscopy (SEM) image of the MFCs with a rectangular GMR element, exhibiting tapered thickness of the MFCs, which naturally formed by the shadow of the photoresist. The tapered shape is reported to be effective in concentrating the magnetic flux toward an MR element [22,24]. Δ*R*/*R*_min_ measurements were carried out at room temperature by connecting the electric current (*I*) and voltage (*V*) probes, as shown in Figure 4b.

## 4. Results and Discussion: FEM Simulations

### 4.1. Rectangular FL

When *B*_ex_ is applied to the GMR sensor with MFCs, the magnetic flux density inside the FL (*B*_FL_) is larger than *B*_ex_ due to the lower magnetic reluctance through the MFCs than that in the air. Thus, the ratio of *B*_FL_/*B*_ex_ represents the figure-of-merit of the MFCs. Therefore, the optimal sensor design is the one which maximizes *B*_FL_/*B*_ex_.

**Figure 5:**
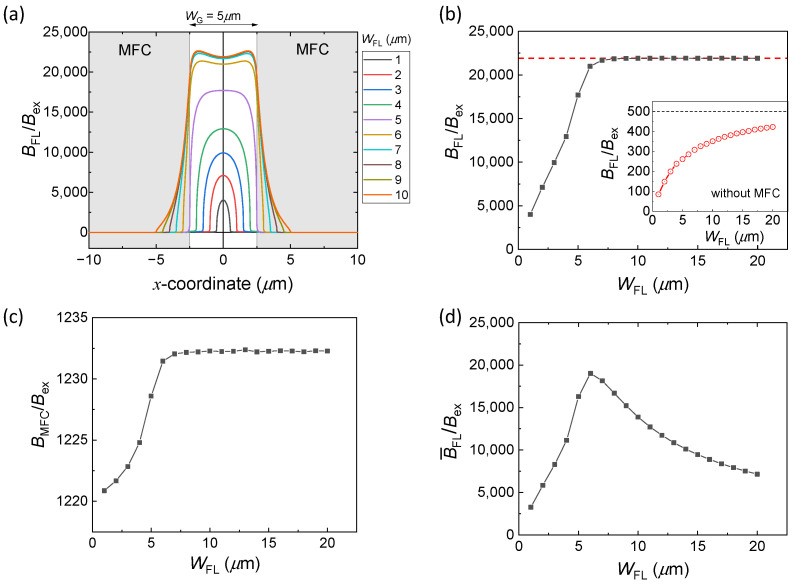
(**a**) *B*_FL_/*B*_ex_ across the line passing through a center of the FL for different *W*_FL_ (*W*_G_ = 5 μm). (**b**) *B*_FL_/*B*_ex_ at the center of the FL for different *W*_FL_. Inset of (**b**) shows *B*_FL_/*B*_ex_ vs. *W*_FL_ without MFCs. (**c**) *B*_MFC_/*B*_ex_ vs. *W*_FL_ curve, showing maximum *B*_MFC_/*B*_ex_ for the cases with an MFC–FL overlap. (**d**) $\bar{B}$_FL_/*B*_ex_ at the center of the FL for *W*_FL_. $\bar{B}$_FL_ is the average magnetic flux density inside the FL defined by Equation (1).

In this section, we discuss the effect of the MFC–FL overlap on *B*_FL_/*B*_ex_. We simulated the distribution of magnetic flux in the FL for different *W*_FL_ (from 1 μm to 20 μm) while keeping the other parameters unchanged. Figure 5a shows the profiles of *B*_FL_/*B*_ex_ along the line parallel to the *x*-direction and passing through the center of the FL. The maximum *B*_FL_/*B*_ex_ was at the center of the FL (represented at *x* = 0) for *W*_FL_ ≤ *W*_G_ (= 5 μm). On the other hand, for *W*_FL_ > *W*_G_, the maximum *B*_FL_/*B*_ex_ was observed near the edges of the MFCs. In addition, for *W*_FL_ > *W*_G_, *B*_FL_/*B*_ex_ rapidly decreased in the region of the FL overlapping with the MFCs (i.e., at *x* > 2.5 μm and *x* < −2.5 μm). This is because the regions of FL under the MFCs are magnetically shielded by the MFCs; thus, only a small magnetic flux flows in those regions. Figure 5b shows *B*_FL_/*B*_ex_ at the center of the FL (*x* = 0) for different *W*_FL_. The inset of Figure 5b shows the *B*_FL_/*B*_ex_ values in the absence of MFCs, which are lower and reaching toward $\mu_{r}^{\;\mathrm{FL}\;}=500$ with an increase in *W*_FL_. This is due to the demagnetization field (*H*_d_) of the patterned FL, which reduces *B*_FL_ as *B*_FL_ = $\mu^{\;\mathrm{FL}}{(H}_{\mathrm{ex}}{\;-\;H}_{d})$. Here, *H*_ex_ (=*B*_ex_/*μ*_0_) is the external magnetic field applied to the sensor. From a practical point of view, we can tell that *H*_d_ in the patterned FL reduces the effective value of $\mu_{r}^{\;\mathrm{FL}}$ from the value in the unpatterned film (in the present case, $\mu_{r}^{\;\mathrm{FL}}=500$).

The value of *B*_FL_/*B*_ex_ in Figure 5b increases as *W*_FL_ increases from 1 μm to 8 μm and reaches the saturation value of 21,900 for *W*_FL_ ≥ 8 μm. This indicates that the MFC–FL overlap enhances *B*_FL_/*B*_ex_ in the middle of the FL because the magnetic reluctance of the sensor is reduced by the MFC–FL overlap as described schematically in Figure 2c. Figure 5c shows the *B*_MFC_/*B*_ex_ vs. *W*_FL_ curve, where *B*_MFC_ is the average magnetic flux density in the MFCs. *B*_MFC_/*B*_ex_ increases with the increase in *W*_FL_ because of the reduction of the overall magnetic reluctance of the system and attains to its maximum value for *W*_FL_ ≥ 8 μm.

From Figure 5a,b, we observe that the MFC–FM overlap enhances *B*_FL_ in the region of the FL under the MFC gap but reduces *B*_FL_ in the other region of the FL. Since the FL in the MFC–FL overlap region receives a much smaller *B*, we lose the output voltage from the GMR element in the region if the length of the MFC–FL overlap region is longer than the optimal value. To understand the gain and loss factors of the MFC–FL overlap, we introduce average magnetic flux density as described below.

The output voltage of a GMR sensor is expressed as ${V=dR/dB}_{x}\cdot B_{\mathrm{FL}}\cdot I$, where ${dR/dB}_{x}$ is the intrinsic sensitivity of the GMR element in the sensing axis (*x*) determined by the MR ratio and the anisotropy field in the FL, *B*_FL_ is the magnetic flux density inside the FL, and *I* is the bias current. ${dR/dB}_{X}$ and the bias current density (*J* in A/m^2^) can be assumed to be uniform in rectangular GMR elements. However, *B*_FL_ is not uniform because of the magneto-static coupling between the FL and MFC and the magnetic shielding effect of the MFC, as shown in Figure 5a. In this case, the output voltage *V* is proportional to the average of *B*_FL_ weighted by the bias current density (*J*). Therefore, we define the average magnetic flux density inside FL, $\bar{B}$_FL_ as follows:

(1) $${\bar{B}}_{\mathrm{FL}}=\frac{ʃʃʃ\;J\left(x,y,z\right)\cdot B\left(x,y,z\right)\;dx\;dy\;dz\;}{ʃʃʃ\;J\left(x,y,z\right)\;dx\;dy\;dz\;}$$

where, the integrations are taken over the whole FL, and *J*(*x*,*y*,*z*) and *B*(*x*,*y*,*z*) represent the bias current density and magnetic flux density at each point of the FL, respectively, with coordinates (*x*, *y*, *z*). Figure 5d shows $\bar{B}$_FL_/*B*_ex_ plotted for *W*_FL_. The maximum $\bar{B}$_FL_/*B*_ex_ of 19,000 was obtained at *W*_FL_ = 6 μm, indicating that the small MFC–FL overlap of only 0.5 μm on both sides of the FL is optimal for the MFC gap *W*_G_ = 5 μm. When *W*_FL_ ≥ 7 μm, $\bar{B}$_FL_/*B*_ex_ rapidly decreased due to the magnetic shielding effect of the MFCs.

Therefore, for rectangular GMR elements with a uniform width *W*_FL_, we found that the MFC–FL overlap (1) enhances *B*_FL_ because the overlapped FL region works as a guide for magnetic flux but (2) loses the output voltage if the MFC–FL overlap is large due to the magnetic shielding effect of the MFCs.

### 4.2. Comb-Shaped FL

**Figure 6:**
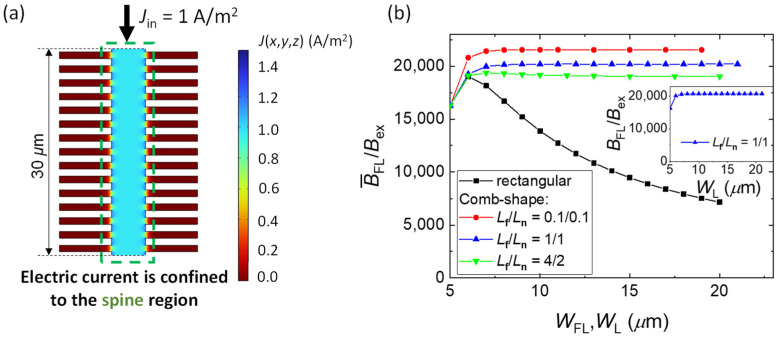
(**a**) Distribution of the electric current density (*J*) in a comb-shaped FL. The notches confine the electric current within the spin region. (**b**) $\bar{B}$_FL_/*B*_ex_ for the comb-shaped FL for different *L*_f_/*L*_n_ combinations of 0.1/0.1, 1/1, and 4/2 (in μm). The inset shows *B*_FL_/*B*_ex_ vs. *W*_L_ of the comb-shaped FL with *L*_f_ = *L*_n_ = 1 μm. $\bar{B}$_FL_/*B*_ex_ of the rectangular FL also plotted for comparison. In the comb-shaped FLs, the MFC–FL overlap does not cause a reduction in $\bar{B}$_FL_/*B*_ex_. Larger $\bar{B}$_FL_/*B*_ex_ can be achieved with a finer comb-shaped FL (i.e., with a smaller *L*_f_ and *L*_n_).

Figure 6a shows the distribution of *J* across the comb-shaped GMR element with *W*_S_ = 5 μm, *W*_L_ = 20 μm, and *L*_f_ = *L*_n_ = 1 μm (a portion of the FL with a length of spine = 30 μm is shown). The electric current is mostly confined in the spine region of the comb-shaped GMR element. Through this effect, the spine region acts as an active GMR element, whereas the fins provide an MFC–FL overlap which helps to increase the magnetic flux flowing into the FL. Figure 6b shows $\bar{B}$_FL_/*B*_ex_ vs. *W*_L_ for the comb-shaped FL with different *L*_f_/*L*_n_ combinations of 0.1/0.1, 1/1, and 4/2 (in μm). The $\bar{B}$_FL_/*B*_ex_ vs. *W*_FL_ values for the rectangular FL are also plotted for comparison. The inset of Figure 6b shows the *B*_FL_/*B*_ex_ value at the center of the FL for different *W*_L_ in the comb-shaped FL with *L*_f_ = *L*_n_ = 1 μm. The *B*_FL_/*B*_ex_ vs. *W*_L_ behavior is very similar to that in the rectangular FL (Figure 5b) and has the saturation value of 20,740 for *W*_FL_ ≥ 8 μm. The small reduction in the saturation value of *B*_FL_/*B*_ex_ in the comb-shaped FL was due to the comparatively smaller area of overlap with the MFCs. However, the behavior of $\bar{B}$_FL_/*B*_ex_ with an increase in *W*_FL_ (i.e., MFC–FL overlap) is different between the comb-shaped FL and the rectangular FL. As mentioned in the previous section, $\bar{B}$_FL_/*B*_ex_ decreased with an increase in the MFC–FL overlap in the rectangular FLs (*W*_FL_ > 6 μm). However, the MFC–FL overlap for the comb-shaped FLs did not cause a reduction in $\bar{B}$_FL_/*B*_ex_. We observed an increase in $\bar{B}$_FL_/*B*_ex_ with *W*_L_ until it attained a saturation value at *W*_L_ = 8 μm, and it retained the same value for a further increase in *W*_L_.

The maximum value of $\bar{B}$_FL_/*B*_ex_ was larger with finer comb shapes, i.e., smaller *L*_f_ and *L*_n_ values. This is because of the more effective current confinement in the spine region for the smaller *L*_f_ and *L*_n_ values. The degree of current confinement and the value of $\bar{B}$_FL_/*B*_ex_ for various shapes of FL are summarized in Table 1. For the comb-shaped FL, various *L*_f_ and *L*_n_ values with a constant *W*_L_ = 15 μm were considered. The data of the rectangular FLs with *W*_FL_ of 5 μm and 15 μm are shown for comparison. The degree of current confinement in the comb-shaped FL is defined as *I*_spine_/*I*_total_, where *I*_spine_ is the current in the spine region and *I*_total_ is the total current flowing in the whole FL. For the rectangular FLs, the current in the region of the FL under the MFC gap (*I*_gap_) is used instead of *I*_spine_. Table 1 also shows the values of $\bar{B}$_FL_/*B*_ex_ without MFC and the MFC gain. Here, the MFC gain is defined as the ratio of the $\bar{B}$_FL_/*B*_ex_ with MFCs to that without MFCs.

The maximum MFC gain of 75 was obtained for the rectangular FL with *W*_FL_ = 5 μm. This is because of the lowest $\bar{B}$_FL_/*B*_ex_ without MFCs for this FL, which is caused by the strong demagnetization field for a narrow *W*_FL_. Despite the largest MFC gain for this sensor, the value of $\bar{B}$_FL_/*B*_ex_, which represents the sensitivity of the sensor, was not the highest for this sensor. The value of $\bar{B}$_FL_/*B*_ex_ with MFCs was larger for the comb-shaped FLs than for the rectangular FLs. In addition, for comb-shaped FLs, higher $\bar{B}$_FL_/*B*_ex_ was obtained for higher *I*_spine_/*I*_total_. For example, *I*_spine_/*I*_total_ of 0.992 was obtained for the comb-shaped FL with *L*_f_ = *L*_n_ = 0.1 μm, indicating that most of the electric current was confined to the spine region of the FL. For this sensor, we obtained the highest $\bar{B}$_FL_/*B*_ex_ of 21,550 with a MFC gain of 63. On the other hand, for the rectangular FL with *W*_L_ = 15 μm, we obtained the lowest $\bar{B}$_FL_/*B*_ex_ of 9470. The data in Table 1 indicate that a maximum MFC gain may not provide a maximum value of $\bar{B}$_FL_/*B*_ex_. This is because MFC gain represents only the change in sensitivity ($\bar{B}$_FL_/*B*_ex_) by an addition of MFCs but does not represent the absolute value of $\bar{B}$_FL_/*B*_ex_. Moreover, the sensitivity of MR sensors with the MFCs depends on factors such as magnetic reluctance, nonuniformity of the magnetic flux density, and the demagnetizing field in the MR element.

Note that the FEM simulation study presented here did not consider magnetization domains in the FLs and MFCs. See the Supplementary Materials for micromagnetic simulations for the representative rectangular and comb-shaped FLs.

## 5. Results and Discussion: Experiments

To validate the above findings with the FEM simulations, we fabricated the GMR SV sensors with rectangular and comb-shaped FLs in combination with MFCs. The rectangular FL had dimensions of *W*_FL_ = 5 and 36 μm and those of the comb-shaped FL were *W*_S_ = 5 μm and *W*_L_ = 36 μm. We compared the GMR ratio (Δ*R*/*R*_min_) and sensitivity, defined as (d*R*/d*H*)/*R*, between the rectangular and comb-shaped FL sensors.

**Figure 7:**
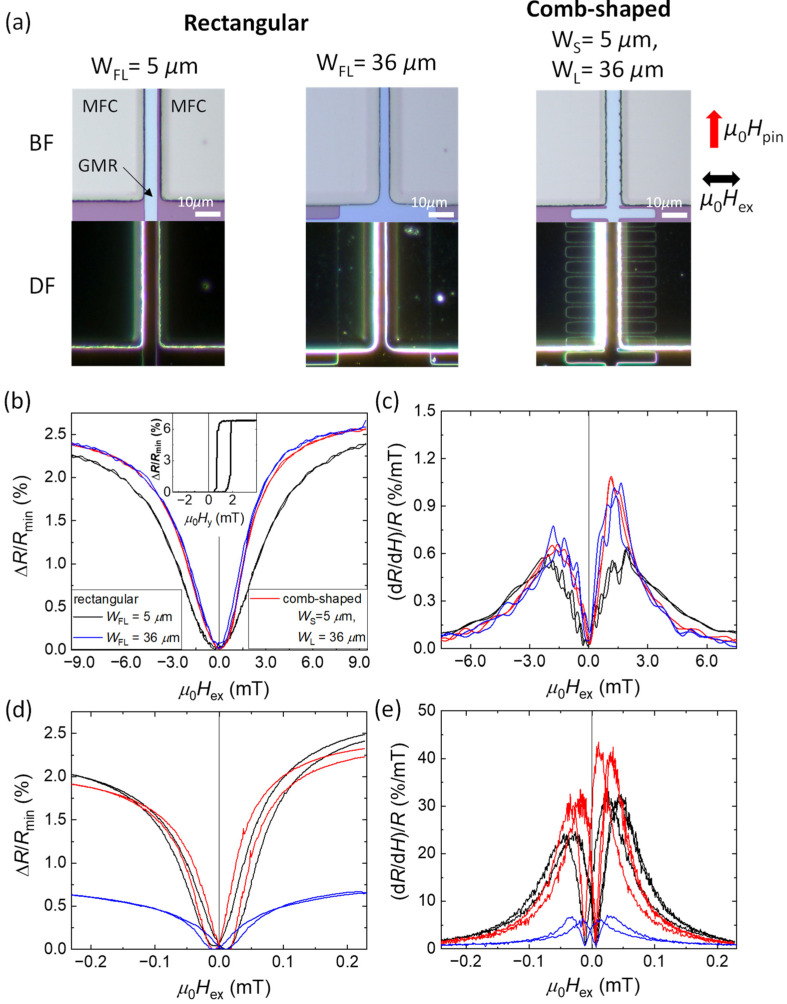
(**a**) Bright field (BF) and dark field (DF) optical microscope images of the rectangular SV with *W*_FL_ = 5 μm and 36 μm, and the comb-shaped SV with *W*_S_ = 5 μm and *W*_L_ = 36 μm. (**b**) Δ*R*/*R*_min_ and (**c**) (d*R*/d*H*)/*R* of the three sensors measured without MFCs. Inset in (**b**) is the Δ*R*/*R*_min_ curve of a rectangular SV with *W*_FL_ = 5 μm for an external magnetic field *μ*_0_*H_y_* in the *y*-direction, i.e., parallel to the pinning direction. (**d**) Δ*R*/*R*_min_ and (**e**) (d*R*/d*H*)/*R* of the same sensors with the MFCs.

Figure 7a shows the bright field (BF) and dark field (DF) optical microscope images of these sensors. Although the simulation results showed that smaller *L*_f_ and *L*_g_ gave larger $\bar{B}$_FL_/*B*_ex_, we made a comb-shaped FL with *L*_f_ = 4 μm and *L*_g_ = 2 μm due to the resolution limit of our photolithography system. The magnetizations of the PL and RL of the SV were pinned in the ∓*y*-directions, respectively. Figure 7b,c show Δ*R*/*R*_min_ and the sensitivity, defined by (d*R*/d*H*)/*R*, of the GMR elements without the MFCs for an external magnetic field (*μ*_0_*H*_ex_) in the *x*-direction, i.e., perpendicular to the pinning direction. The inset of Figure 7b shows the Δ*R*/*R*_min_ curve of a rectangular SV with *W*_FL_ = 5 μm for an external magnetic field *μ*_0_*H_y_* in the *y*-direction, i.e., parallel to the pinning direction. Δ*R*/*R*_min_ vs. *μ*_0_*H_y_* shows Δ*R*/*R*_min_~6.9% for a complete rotation of the FL from the +*y* to −*y* direction. Moreover, a shift of 1.1 mT in Δ*R*/*R*_min_ vs. *μ*_0_*H_y_* is observed from *μ*_0_*H_y_* = 0, giving Δ*R*/*R*_min_ = 0 at *μ*_0_*H_y_* = 0. This indicates that the FL magnetization is weakly stabilized in the +*y*-direction, probably through an orange-peel ferromagnetic coupling between RL and FL. Due to this ferromagnetic coupling, the magnetization of FL remains parallel to that of RL in the absence of an external magnetic field. Therefore, this configuration gave even-function type *R*-*H* curves in *x*-direction with minimum resistance at zero field. These devices with the even-function *R*-*H* response were intended for use with the AC magnetic field modulation for reduction of 1/*f* noise [18,27]. At *μ*_0_*H* = 10 mT, we obtained Δ*R*/*R*_min_~2.3%, where the FL magnetization was nearly saturated in the *x*-direction. The asymmetries of the Δ*R*/*R*_min_ and (d*R*/d*H*)/*R* curves with respect to *μ*_0_*H* = 0 were derived from a misalignment of the direction of the magnetic field during annealing from the *y*-direction.

For the both comb-shaped SVs and rectangular SV with *W*_FL_ = 36 μm, the maximum value of sensitivity (d*R*/d*H*)/*R* without MFCs was 0.7 %/mT and 1 %/mT for negative and positive *H*, respectively, resulting in the average maximum sensitivity (*S*_max_) of 0.85 %/mT. On the other hand, the rectangular SV with a smaller *W*_FL_ = 5 μm showed a *S*_max_~0.6 %/mT. The smaller sensitivity for the smaller *W*_FL_ is due to the larger demagnetizing field in the *x*-direction.

Figure 7d,e show Δ*R*/*R*_min_ and (d*R*/d*H*)/*R*, respectively, of the same sensors after fabrication of the MFCs. All sensors showed *μ*_0_*H*_c_ of 0.02 mT, which might be derived from that of the MFCs. The rectangular sensor with *W*_FL_ = 36 μm showed the lowest Δ*R*/*R*_min_ due to the loss of the GMR by the MFC–FL overlap, as discussed in Section 4.1, resulting in *S*_max_~6 %/mT. On the other hand, the comb-shaped SV and the rectangular SV with *W*_FL_ = 5 μm maintained Δ*R*/*R*_min_ above 2.0% after fabrication of the MFCs. However, the sensitivity was higher for the comb-shaped SV: *S*_max_~36.5 %/mT (31 %/mT and 42 %/mT for negative and positive *H*, respectively). On the other hand, the rectangular SV with *W*_FL_ = 5 μm exhibited *S*_max_~28 %/mT. The *S*_max_ and MFC gains of these sensors with and without MFCs are summarized in Table 2. The MFC gains of the rectangular SV with *W*_FL_ = 5 μm and *W*_FL_ = 36 μm and the comb-shaped SV were 47, 7, and 43, respectively. The highest MFC gain for the rectangular SV with *W*_FL_ = 5 μm was a result of its lowest sensitivity (~0.6 %/mT) without the MFCs owing to a stronger demagnetization field for a smaller *W*_FL_. Due to this, the effectiveness of the MFCs in reducing *H*_d_ was greater in the rectangular SV with *W*_FL_ = 5 μm. However, as mentioned in the previous section, the maximum MFC gain does not necessarily give the maximum sensitivity. The sensitivity was the highest in the comb-shaped SV owing to the low magnetic reluctance caused by the magneto-static coupling between the fins and MFCs as shown by the FEM simulations.

These results demonstrate that MFC gain is not a sufficient parameter to describe the effect of the MFC. For the appropriate design of MR sensors combined with MFCs, the magnetic reluctance of the whole sensor system (i.e., the MR element and MFCs) and the nonuniformity of the magnetic flux density in the MR element should be considered.

## 6. Conclusions

We studied the effect of the geometrical overlap between the MFCs and FL (MFC–FL overlap) of GMR sensors using FEM simulations and experiments. We observed that the MFC–FL overlap enhances the magnetic flux density in the center of the FL, i.e., the region of the FL under the MFC gap. However, the magnetic flux density in the region of the FL overlapped with the MFC decreases due to the magnetic shielding effect of the MFC, by which the MR ratio of the GMR sensor was decreased in the conventional rectangular-shaped GMR. To quantify the effect of the MFC–FL overlap on the sensitivity of the sensor, we defined the effective magnetic flux density, $\bar{B}$_FL_, which incorporates the effect of nonuniformity in the magnetic flux density on the output voltage of the GMR sensor.

To overcome the reduction of output voltage in the case of the rectangular FLs overlapped with the MFCs, we propose a comb-shaped GMR sensor with alternate fins and notches. The notches confine the electric current in the spine region of the comb-shaped GMR sensor, and the fins provide the MFC–FL overlap. Using this shape, we obtained a strong magneto-static coupling between the MFC and GMR sensor without losing its Δ*R*/*R*_min_. The advantage of the comb shape in comparison with the conventional rectangular GMR sensor has been demonstrated experimentally. We obtained a sensitivity of 36.5 %/mT for the comb-shaped GMR sensor as compared to 28 %/mT for the rectangular GMR sensor. This study shows that the magnetic reluctance and the nonuniformity of the magnetic flux density in MR elements should be considered when designing MR sensors with MFCs.

## Figures and Tables

**Table 1:** Degree of confinement of the electric current, $\bar{B}$_FL_/*B*_ex_ with and without MFC, and MFC gain in different FLs with a constant *W*_L_ = 15 μm and different *L*_f_ and *L*_n_. The values for the rectangular FL with *W*_FL_ = 5 and 15 μm are also given for comparison. For simulations, the MFC gap was fixed to *W*_G_ = 5 μm.

FL Shape (in μm)	Degree of Confinement of Electric Current (*I*_spine_/*I*_total_)	${\bar{B}}_{FL}/B_{ex}$ with MFC	${\bar{B}}_{FL}/B_{ex}$ without MFC	MFC Gain
Comb shape, *W*_L_ = 15 *L*_f_ = *L*_n_= 0.1	0.992	21,550	340	63
Comb shape, *W*_L_ = 15 *L*_f_ = *L*_n_ = 1.0	0.952	20,200	338	60
Comb shape, *W*_L_ = 15 *L*_f_ = 4, *L*_n_ = 2	0.787	19,050	359	53
Rectangular *W*_FL_ = 5	(*I*_gap_/*I*_total_ =) 1.0	16,300	218	75
Rectangular *W*_FL_ = 15	(*I*_gap_/*I*_total_ =) 0.333	9470	345	27

**Table 2:** Summary of the maximum sensitivity values and MFC gains of the rectangular and comb-shaped GMR sensors with and without MFCs. −*S*_max_ and +*S*_max_ indicate the maximum sensitivities measured for negative and positive *H*, respectively (see Figure 7).

Device	Max Sensitivity without MFC (%/mT)			Max Sensitivity with MFC (%/mT)			MFC Gain
	−*S*max	+*S*max	Avg. *S*max	−*S*max	+*S*max	Avg. *S*max	
Rectangular *W*_FL_ = 5	0.6	0.6	0.6	24	32	28	47
Rectangular *W*_FL_ = 36	0.7	1	0.85	6	6	6	7
Comb shape *W*_L_ = 36, *W*_S_ = 5	0.7	1	0.85	31	42	36.5	43

## Data Availability

The data supporting the findings of this study are available from the corresponding author upon reasonable request.

## Supplementary Materials

The following supporting information can be downloaded at https://www.mdpi.com/article/10.3390/s22239385/s1.
