# Supplementary material for: The Effect of Geometrical Overlap between Giant Magnetoresistance Sensor and Magnetic Flux Concentrators: A Novel Comb-Shaped Sensor for Improved Sensitivity
Source: Sensors (Basel). 2022 Dec 1;22(23):9385. doi: 10.3390/s22239385 (PMC9740504; doi:10.3390/s22239385)
Supplement: Supplementary file 1 [file sensors-22-09385-s001.zip › sensors-2043889-supplementary.pdf]

Supplementary Material

Micromagnetic simulations of magnetization distribution in rectangular and comb-shaped magnetic free layers

We studied the effect of the shape of FL on its magnetization distribution by the Object Oriented MicroMagnetic Framework (OOMMF). For the micromagnetic simulations, a single layer of ferromagnetic material is considered as the free layer (FL) of a spin valve (SV) GMR. We defined the FL of a material with a saturation magnetization ($\mu_0 M_s$) of 1 T and a negligible magneto-crystalline anisotropy. The simulations were carried out on three types of FLs (see Fig. S1): (i) rectangular FL with W_{FL} = 5 μm, (ii) rectangular FL with W_{FL} =10 μm, (iii) comb-shaped FL with W_S = 5 μm, W_L= 10 μm, and L_f = L_n = 1 μm. The length (in y-direction) and thickness (in z-direction) of all three FLs were kept to 100 μm and 10 nm, respectively. A constant magnetic bias field of 1 mT was applied to FLs in the +y-direction to imitate the ferromagnetic interlayer coupling between the FL and RL of the SV which was observed in our experiments (see Section 5). We simulated the effect of external magnetic field on the magnetization distribution of the FLs by applying $\mu_0 H_{ex}$ of 0 mT to 5 mT along the +x-direction, as shown in Fig. S1. We defined an angle θ as the angle between the bias field $\mu_0 H_y$ and the average magnetization ($\mu_0 M$) of the FL. We did not consider MFCs in these simulations.

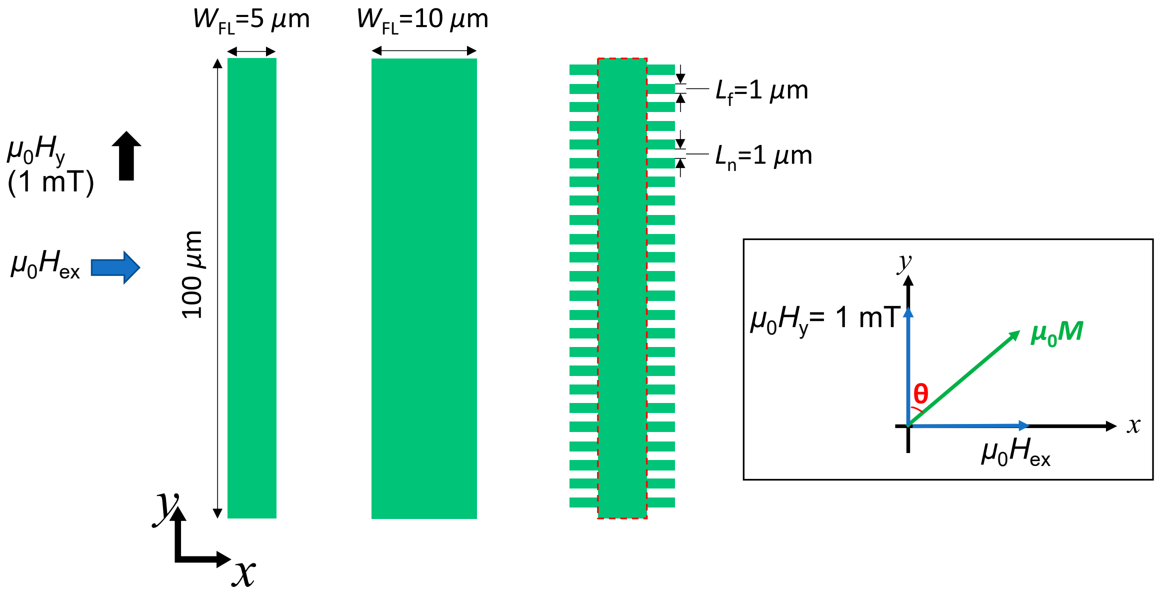

Figure S1. Schematic diagram of three FLs: (i) rectangular FL with W_{FL} = 5 μm, (ii) rectangular FL with W_{FL} =10 μm, and (iii) comb-shaped FL with W_S = 5 μm, W_L= 10 μm, and L_f = L_n = 1 μm. The length (in the y-direction) and thickness (in z-direction) of all three FLs were kept to 100 μm and 10 nm, respectively. The micromagnetic simulations were carried out to calculate the angle (θ) between the bias field $\mu_0 H_y$ and the average magnetization ($\mu_0 M$) of FL. For the comb-shaped FL, the average magnetization of only the spine region (*i.e.*, the active region of MR) was considered.

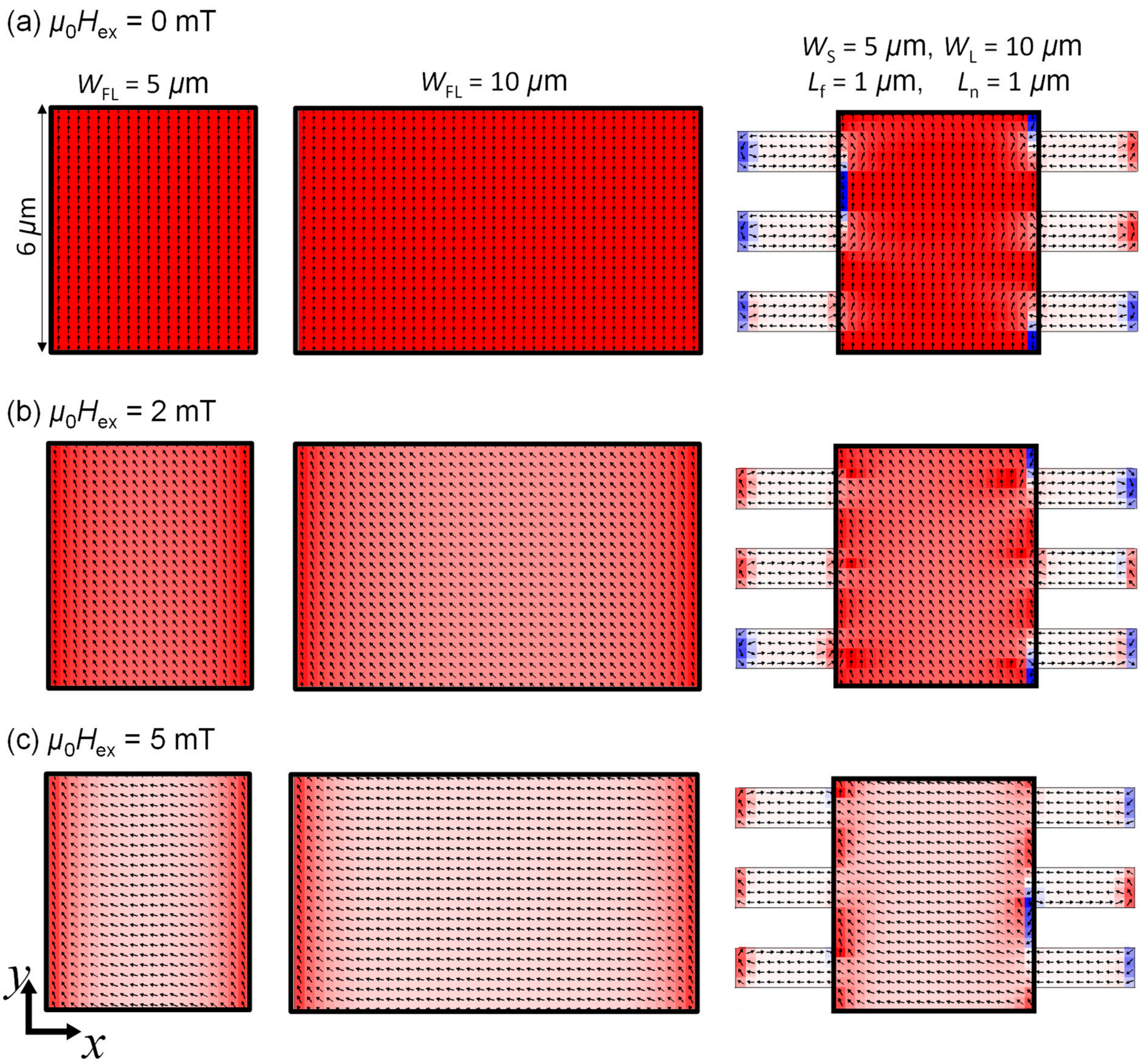

Figure S2. Micromagnetic simulations of the rectangular FL with W_{FL} = 5 μm and 10 μm, and the comb-shaped FL with W_S = 5 μm and W_L= 10 μm (L_f = L_n = 1 μm). (a), (b), and (c) show the magnetization at the external field of μ_0H_{ex} = 0 mT, 2 mT, and 5 mT, respectively. The arrows indicate the magnetization direction in the *xy*-plane. The color of pixels represents a *y*-component of the magnetization with red, white, and blue colors corresponding to the *y*-component of +μ_0M_S, 0, and -μ_0M_S, respectively.

Figure S2 shows the magnetization across the central region of three FLs (length in *y*-direction is 6 μm) under different μ_0H_{ex} of 0 mT, 2 mT, and 5 mT. The arrows represent the direction of magnetization at each point of the FL. The color of the pixels indicates the *y*-component of the magnetization at that position (red, white, and blue represent +μ_0M_s, 0, and −μ_0M_s, respectively). At μ_0H_{ex} = 0, the magnetization across the FL was aligned in the +*y*-direction in both rectangular FLs due to the bias field of 1 mT. However, this was not the case with the comb-shaped FL. The magnetization in the fins was demagnetized (*i.e.*, net magnetization in the *x* and *y* directions ~0) in the ±*x*-directions (see Fig. S2 (a)). Nevertheless, the magnetization in the spine region of the comb-shaped FL was aligned in the +*y*-direction, similar to the magnetizations of the rectangular FLs. With an application of μ_0H_{ex}, the magnetization of the rectangular FL and that of the spine region of the comb-shaped FL started rotating toward μ_0H_{ex} (see Figs. S2 (b) and (c)). To visualize the rotation of the magnetization with μ_0H_{ex}, we calculated the angle θ between μ_0H_y and the average magnetization, μ_0M, at different μ_0H_{ex}. For the comb-shaped FL the average

magnetization of only the spine region (*i.e.*, the active region of MR) was considered since only the spine region contributes to the output voltage.

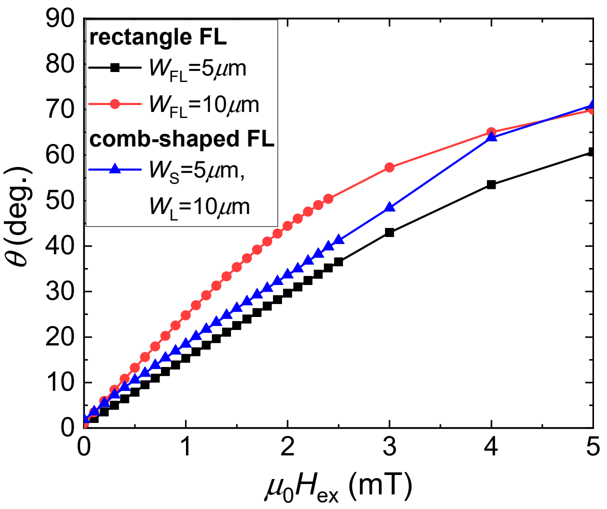

Figure S3. θ vs. $\mu_0 H_{ex}$ in three different FLs: the rectangular FLs with W_{FL} = 5 μm and 10 μm, and the comb-shaped FL with W_S = 5 μm, W_L= 10 μm, and L_f = L_n = 1 μm.

Figure S3 shows the graph of θ vs. $\mu_0 H_{ex}$ for the three types of FLs. θ ~ 0° at $\mu_0 H_{ex}$ = 0 mT in all three FLs and θ increased monotonically with the increase in $\mu_0 H_{ex}$, approaching toward 90° (*i.e.*, parallel to $\mu_0 H_{ex}$). At a given value of $\mu_0 H_{ex}$ (up to 5 mT), θ was the smallest for the rectangular FL with W_{FL} = 5 μm due to the larger demagnetization field in the narrower FL. This indicates that this FL has a lower sensitivity to $\mu_0 H_{ex}$ compared with the other FLs. In the comb-shaped FL, the addition of the fins reduces the demagnetization field and thus increases θ (*i.e.*, increased sensitivity to $\mu_0 H_{ex}$). However, θ remains smaller than the rectangular FL with W_{FL} = 10 μm.

In summary, the following observations can be drawn from the micromagnetic studies:

（1）Magnetizations in the spine region and fin region of the comb-shaped FL were not parallel at $\mu_0 H_{ex}$ = 0. The magnetization in the spine region remained parallel to the bias field $\mu_0 H_y$, whereas that in the fin region was in the demagnetized state.

（2）Nevertheless, the addition of fins increased the sensitivity (*i.e.*, larger θ at given $\mu_0 H_{ex}$) of the spine region to the external field $\mu_0 H_{ex}$.

（3）The sensitivity of the FL magnetization to $\mu_0 H_{ex}$ was expected to be the largest for the rectangular FL with width W_{FL} = 10 μm. However, as discussed in the paper, the presence of the MFC–FL overlap in the rectangular FL with large W_{FL} caused a reduction in the output signal due to a shunting of the magnetic flux by the MFC.
